# Atypical coordination of cortical oscillations in response to speech in autism

**DOI:** 10.3389/fnhum.2015.00171

**Published:** 2015-03-27

**Authors:** Delphine Jochaut, Katia Lehongre, Ana Saitovitch, Anne-Dominique Devauchelle, Itsaso Olasagasti, Nadia Chabane, Monica Zilbovicius, Anne-Lise Giraud

**Affiliations:** ^1^Department of Neurosciences, University of GenevaGeneva, Switzerland; ^2^Centre de Recherche de l’Institut du Cerveau et de la Moelle Epinière, INSERM UMRS 975 – CNRS UMR 7225, Hôpital de la Pitié-SalpêtrièreParis, France; ^3^Unité Inserm 1000, Service de Radiologie Pédiatrique, Hôpital Necker – Enfants-Malades, AP-HP, Université Paris V René-DescartesParis, France; ^4^Inserm U960, Département des Etudes Cognitives, Ecole Normale SupérieureParis, France; ^5^Unité Multidisciplinaire pour la Santé des Adolescents, Centre Cantonal de l’Autisme, Centre Hospitalier Universitaire VaudoisLausanne, Switzerland

**Keywords:** speech processing, auditory cortex, cortical oscillations, oscillation coupling, autism

## Abstract

Subjects with autism often show language difficulties, but it is unclear how they relate to neurophysiological anomalies of cortical speech processing. We used combined EEG and fMRI in 13 subjects with autism and 13 control participants and show that in autism, gamma and theta cortical activity do not engage synergistically in response to speech. Theta activity in left auditory cortex fails to track speech modulations, and to down-regulate gamma oscillations in the group with autism. This deficit predicts the severity of both verbal impairment and autism symptoms in the affected sample. Finally, we found that oscillation-based connectivity between auditory and other language cortices is altered in autism. These results suggest that the verbal disorder in autism could be associated with an altered balance of slow and fast auditory oscillations, and that this anomaly could compromise the mapping between sensory input and higher-level cognitive representations.

## Introduction

Expressive and receptive language difficulties are frequently observed in autism and have long been a diagnostic symptom. The reason why children with autism inadequately respond to the speech of their closest relatives remains unexplained, presumably because (i) the genetic pattern in autism involves a complex combination of genetic and epigenetic factors (Peñagarikano et al., 2011; Peñagarikano and Geschwind, 2012; Murdoch and State, 2013; Pu et al., 2013); (ii) there is no consensus about how genetically altered corticogenesis could impact collective neuronal functioning and cognitive operations; and (iii) the neural mechanisms of speech processing are only partially understood. According to the DSM5 (American Psychiatric Association [APA], 2013), subjects with ASD exhibit “hyper- or hypo-reactivity to sensory input," which could mean that speech and language deficits in autism reflect auditory (Edgar et al., 2013; Kujala et al., 2013) rather than (or in addition to) higher-level linguistic dysfunctions (Stevenson et al., 2014a).

We explored whether subjects with autism exhibit a neurophysiological deficit in speech processing (Eyler et al., 2012), basing some of our hypotheses on recent advances on the role of cortical oscillations in speech segmentation and decoding (Ghitza and Greenberg, 2009; Ghitza, 2011; Giraud and Poeppel, 2012; Gross et al., 2013; Doelling et al., 2014). In autism, accelerated neocortical maturation (Courchesne et al., 2003) co-occurs with laminar disorganization in the temporal cortex (Jacot-Descombes et al., 2012) where it compromises the development of auditory and language micro- and macro-circuits (Eyler et al., 2012; Williams et al., 2013). Because cortical oscillations arise from laminar-specific interactions between excitatory and inhibitory neurons (Rotstein et al., 2005; Ainsworth et al., 2011; Whittington et al., 2011), the migration anomalies and local alterations of GABA inhibition observed in autism (Bartos et al., 2007; Tyzio et al., 2014) could directly interfere with the generation of important neurophysiological response patterns such as theta and gamma oscillations, preventing them from playing their expected parsing and decoding roles in speech processing. During development, such anomalies could delay language acquisition, because speech would evoke less reliable neural temporal patterns (Dinstein et al., 2012), compromising the interfacing between auditory cortex and the rest of the language network/other cognitive systems (Uhlhaas and Singer, 2007).

The syllabic structure of speech engages auditory cortical responses in the theta (4–7 Hz) frequency (Luo and Poeppel, 2007; Ghitza and Greenberg, 2009; Ding and Simon, 2013), and theta modulations typically influence gamma signals through nesting, a mechanism whereby the energy in gamma activity is controlled by the phase of theta activity (Schroeder et al., 2008). It is assumed that theta/gamma nesting enables speech decoding by orchestrating neural activity into a syllable-based code aligned on key phonemic events (Ghitza, 2011; Giraud and Poeppel, 2012). Critically, this model predicts that speech decoding is compromised if theta activity fails to track speech modulations (Ahissar et al., 2001; Luo and Poeppel, 2007; Ghitza, 2012) and to shape gamma activity (Giraud and Poeppel, 2012). Accordingly, reduced reactivity to voice modulations in autism (Gervais et al., 2004; Abrams et al., 2013) suggests a speech tracking dysfunction reflected in auditory theta activity. A consequence of this anomaly would be reduced down-regulation of gamma by theta activity, and less accurate speech parsing and encoding. Here we test the processing of speech by participants with autism and controls by recording concurrent EEG and fMRI data while they viewed an engaging documentary film.

## Materials and Methods

### Participants

Thirty-one subjects (adults and adolescents) participated in a combined EEG/fMRI study. Fifteen of these were identified as presenting with primary autism disorder with language impairments, diagnosed according to DSM-IV criteria, and further confirmed with the Autism Diagnostic Interview-Revised (Lord et al., 1994). We excluded subjects with infectious, metabolic, neurological, or genetic diseases, abnormal hearing levels, and those who were unable to stay confined and still in the MRI scanner. All subjects and their legal representative(s) provided written informed consent for participation in the study, which was approved by the local ethics committee (Biomedical Inserm protocol C08-39). We collected IQ measures (short form of the WAIS-III scale, Weschler, 2000) in all subjects, and autism-spectrum quotients (AQs, Baron-Cohen et al., 2001) and the verbal component of the Autism Diagnostic Interview-Revised in all but three (one deeply dysphasic, one moderately dysphasic, and one for whom the parents did not give consent to the tests). The two ASD subjects with expressive difficulties were not taken into account in the statistics involving clinical data. These subjects are shown on the related figures, in order for readers to assess their relation to the group. Psychometric data are summarized in **Table 1**. Because we focused on low-level properties of auditory cortex as a possible basic sensory dysfunction in autism, we did not restrict our observations to the high IQ subpopulation with autism (Asperger) or to any specific autism subprofile.

**Table 1:** Psychometric data.

Group	Age	IQ	AQ	ADIb	ADInvc	ADIvc	ADId	Total ADI
ASD	20	50	Profoundly dysphasic	16	12	–	7	35
ASD	27	90	–	24	11	17	8	43
ASD	20	82	Moderately dysphasic	13	7	–	6	26
ASD	22	79	26	20	11	15	2	33
ASD	19	110	28	23	11	17	4	38
ASD	17	66	28	28	11	17	5	44
ASD	15	80	21	17	5	12	8	30
ASD	17	124	33	22	14	21	11	47
ASD	15	35	26	23	5	16	10	38
ASD	16	85	43	20	14	23	3	37
ASD	17	120	21	25	17	22	2	44
ASD	40	75	32	36	14	21	7	57
ASD	17	91	27	25	14	18	5	44
Control	20	106	19					
Control	27	97	20					
Control	19	97	13					
Control	23	102	14					
Control	16	114	12					
Control	20	105	6					
Control	17	112	12					
Control	18	123	9					
Control	17	92	11					
Control	38	94	7					
Control	20	97	14					
Control	40	95	10					
Control	16	127	9					

*ADI, Autism Diagnostic Interview; ADIb, stereotyped behavioral component of the Autism Diagnostic Interview; ADInvc, non verbal component of the Autism Diagnostic Interview; ADIvc, verbal component of the Autism Diagnostic Interview; ADId, social component of the Autism Diagnostic Interview; AQ, Autism-Spectrum Quotient; IQ, intellectual quotient*.

### Experimental Procedure

We explored auditory cortical processing during a passive, naturalistic task with a relatively low cognitive demand while both EEG and fMRI were being concurrently recorded. Subjects viewed a TV program for youth, selected to engage the interest of participants with ASD. The program was an audio-visual scientific documentary about the dangers of the sun on seashores (see **Movie S1**), featuring three different speakers (two males) who made scientific demonstrations while talking to the audience, and occasionally to each other. Participants were asked to rest with eyes closed (movie off), or to watch the movie, in alternation, for short periods of 5 min, over three sessions (sessions one and two: 5 min of movie followed by 5 min of rest, and session three: 5 min of rest). To minimize the influence of the movie on the following resting state period we only analyzed the last 4.5 min of rest. The subjects were instructed to attentively watch the program and were informed that they would have to give a brief report about its content after the MRI sessions. They were also instructed to refrain from moving or falling asleep during the resting periods. Attention was controlled using EEG monitoring of the alpha rhythms and in some subjects by eye tracking. We also used EEG to track movement artifacts, and excluded three of the 31 subjects who exhibited more than one movement artifact per minute. We had to exclude two other subjects due to technical problems during the recordings (malfunction of the sound system and of the amplifier). The remaining 26 participants were comprised of 13 subjects with autism (mean age = 20.67 ± 6.77 years, mean IQ = 83.61 ± 25.27) and 13 control participants (mean age = 22.92 ± 8.14, mean IQ = 104 ± 11.28) matched for age and not for IQ (**Table 1**). This sample size remains theoretically sufficient to detect medium to large effect sizes (Friston, 2012).

At the end of the scanning sessions, subjects were asked to report what the TV program was about and what the speakers’ names were. All participants except for two subjects with autism (the two subjects with dysphasia) correctly reported that the movie was about the dangers of sun exposure, and correctly provided the names of the main speakers. The two subjects who did not provide satisfactory answers were excluded from analyses involving clinical variables, and were included in the neurophysiological analyses only after verification that they were not outliers [Grubbs’ test for the theta and gamma parameter estimate variables, theta: mean = 0.017, SD = 0.035, *G*(0.05) < 2.84; gamma: mean = -0.0017, SD = 0,109, *G*(0.05) < 2.84]. The neurophysiological effects were then related to clinical variables (AQ, ADI verbal communication component), while the non-verbal communication component served as a control variable.

### MRI and EEG Acquisition and Preprocessing

Six hundred eighty echoplanar fMRI image volumes (Tim-Trio; Siemens, 40 transverse slices, voxel size = 3 mm × 3 mm × 3 mm; repetition time = 2,000 ms; echo time = 50 ms; field of view = 192) were acquired during the first two sessions, and 310 image volumes during the third one. Continuous EEG was simultaneously recorded with a 5 kHz sampling rate from 12 scalp sites (Easycap electrode cap, International 10–20 system: F3, F4, C3, C4, T7, T8, P3, P4, O1, O2, reference in Cz, Ground in AFz) using MR compatible amplifiers (BrainAmp MR and Brain Vision Recorder software; Brainproducts). One additional electrode for the electrocardiogram was placed under the left shoulder blade. Impedances were kept under 10 kΩ, and EEG was time-locked with the scanner clock, which further reduced artifacts and resulted in higher EEG quality in the gamma band. A 7-min anatomical T1-weighted magnetization-prepared rapid acquisition gradient echo sequence (176 slices, field of view = 256, voxel size = 1 mm × 1 mm × 1 mm) was acquired at the end of scanning.

We used statistical parametric mapping (SPM8; Wellcome Department of Imaging Neuroscience, UK^1^) for fMRI standard preprocessing (realignment, coregistration with structural images, segmentation, and normalization in the Montreal Neurological Institute stereotactic space). The images were spatially smoothed using a 10-mm full-width half-maximum isotropic Gaussian kernel. Gradient and pulse artifacts were first detected and then marked using in-house software^2^ that correlated the data with automatically (for gradient) or manually (for pulse) defined templates. Artifacts were corrected using PCA, using FASST v111017^3^ for gradient artifacts, and EEGLab v0.9^4^ for pulse artifacts. We excluded F3 and F4 from the analyses, as this pair of electrodes mostly captures the frontal eye field (Amiez and Petrides, 2009). Data were subsequently down-sampled to 250 Hz and re-referenced to a common average reference. The original reference electrode was recalculated as FCz, resulting in a total of 13 cortical electrodes. For each subject, periods with head movement artifacts were detected by visual inspection, and excluded as described in the EEG informed-fMRI section.

### Analyses of the fMRI Dataset

We first analyzed the fMRI data set alone, using a general linear model (GLM) implemented in SPM8. We initially assessed whole brain activity at the single-subject level. The Gaussian distribution of the data allowed us to perform parametric tests. We included motion parameters and their first and second derivatives, the averaged signal of three separate brain compartments (white-matter, gray-matter, and CSF), and all out-of-brain voxels as nuisance covariates. In a second step, we selectively explored speech-related cortical responses by modeling the acoustic envelope of the speech part of the audiovisual sequence in the statistical analysis. The speech envelope was obtained by calculating the Hilbert transform of the stimuli and then filtering the magnitude of the result with a passband of 2–30 Hz. We verified for outliers showing task-related motion artifacts^5^, and further minimized spurious effects of head motion (Chase, 2014) by modeling head motion parameters and their first and second derivatives as covariates of no interest.

Contrast images (movie/rest) were created for each subject and entered into a second level analysis in which IQ was used as a nuisance variable (covariate). As the variance between the two groups was unequal, group differences between subjects with and without autism were assessed using 2-tailed two-sample *t*-tests for each condition. Each group comparison was masked by the relevant main effect of group. Due to a priori predictions of findings within Heschl’s gyrus, we performed small volume corrections (SVCs) on the results within this region. The SVC was done using an independently defined region of interest, anatomically defined with the aal atlas (implemented in xjview^6^). False positives in auditory cortex were further eliminated using an extend threshold >30 voxels for all analyses. For display purposes, we show whole-brain uncorrected statistics. All brain maps are displayed using MRIcron software^7^.

### EEG-Informed fMRI

In a second step, we used combined fMRI and EEG to measure power fluctuations of rhythmic cortical activity and its topography in subjects with and without autism spectrum disorder. We used this approach to localize regions where blood oxygen-level dependent (BOLD) fluctuations systematically covary with EEG power fluctuations (Laufs et al., 2006; Giraud et al., 2007; Morillon et al., 2010). While the BOLD effect reflects overall synaptic activity (Logothetis, 2010), cortical oscillations – and in particular theta and gamma oscillations, as recorded with EEG, primarily denote activity involving pyramidal cells (Buzsáki et al., 2012). By combining the two recording techniques we determine the fraction of the BOLD effect that is linked to pyramidal cell activity, at theta and gamma rhythms, which are hypothesized to underpin speech parsing and syllable encoding (Giraud and Poeppel, 2012).

We used EEG power fluctuations in specific frequency bands of interest (averaged for theta over 4–7 Hz, and for low gamma over 30–40 Hz) to inform the fMRI analysis using a GLM. We performed time-frequency (TF) analyses on the EEG signal using a Morlet wavelets approach (Fieldtrip^8^). The TF structure of signals was computed at each channel for frequencies from 1 to 70 Hz, with a frequency step of 1 Hz and a time step of 0.1 s. The power time course of each channel and each frequency was converted to *Z*-scores after replacing values of previously detected periods of movement by NaNs (Not a Number). We removed further residual artifacts by also rejecting *Z*-values above 4. The transformed signal was then averaged over channels, Z-transformed a second time and NaNs were replaced by zeros. Finally, we averaged the transformed signal across frequencies and channels (but F3 and F4), and we used this signal in the subsequent EEG/fMRI analyses. This procedure is state-of-the-art and prevents the issue of having to make source inferences prior to the correlation with fMRI (Laufs et al., 2006). The log-transformed data were normally distributed, which allowed us to use standard parametric statistical tests (for example, paired *t*-tests and Pearson’s correlations).

As both the theta- and gamma-informed MRI models showed significant effects in left auditory cortex during movie viewing, we assessed gamma and theta oscillations engagement during movie viewing (rest vs. movie) in each group in this region. We extracted the parameter estimates for each subject and each condition from the two regions where there were significant group effects at rest, and ran a two-way ANOVA (group × condition) for each model (theta and gamma).

### fMRI-Informed EEG (Partial Correlations)

The previous analysis required that we specify frequency bands of interests. To establish the frequency specificity of the effects found with EEG-informed fMRI for the gamma and theta bands, we explored EEG-BOLD coupling across the whole EEG spectrum in the left auditory region that was more activated in control than ASD subjects during the movie in the fMRI-only analysis. We also explored this coupling in the left visual region that was over-activated during the movie (fusiform gyrus) as a control for the specificity of auditory effects.

For both these regions, we correlated the BOLD time course with EEG power fluctuations across the 1–70 Hz spectrum [resulting from the TF analyses and convolved with the hemodynamic response function (HRF) after concatenation of the three-rest or two-movie sessions]. We modeled head-motion parameters, their derivatives, the averaged signal of white-matter, gray-matter and CSF and out-of-brain voxels as covariates of no interest. Resulting correlation values were Fisher Z-transformed, and standard statistics were performed on a near Gaussian population.

### Correlation of Neurophysiological and Clinical Variables

We assessed the covariation of theta and gamma informed-BOLD responses in the left auditory cortex (and in the right auditory cortex as a control), where we detected a group difference in both theta and gamma models. We tested for a dependence of gamma and theta activity in each group using the Pearson’s correlation test. For each hemisphere, we then performed a univariate analysis of covariance (ANCOVA) with gamma-BOLD parameter estimates as the dependent factor and theta-BOLD parameter estimates as covariates (as we assume gamma activity to be controlled by theta activity). We used the theta × gamma interaction term to test for correlations (Pearson’s correlation test) with clinical variables (AQ, Baron-Cohen et al., 2001, the verbal component of the ADI-R and the non-verbal communication component of the ADI-R). Finally we addressed whether the relation between the theta–gamma interaction variable and the AQ was different between groups, using an ANCOVA with AQ as the dependent factor and theta-gamma variable as a covariate. All analyses were carried out with SPSS (IBM Corp. Released 2011. IBM SPSS Statistics for Windows, Version 18.0., Armonk, NY, USA).

### Oscillation-Based Connectivity Analyses

Finally, we explored oscillation-based connectivity (Morillon et al., 2010) within the language network in each hemisphere. The underlying assumption is that the broad-spectrum oscillatory pattern at rest in one region determines the oscillatory pattern during movie viewing in another region only if the two regions interact functionally by exchanging information in specific frequency bands (Fries, 2009; Morillon et al., 2010). We assessed the degree of similarity of EEG power-BOLD broad-spectrum between rest and movie across nine cortical language regions. The primary motor regions (BA4a and BA4p), the planum temporale (Wernicke’s region: Te3), the ventral prefrontal cortex (Broca’s region: BA44 and BA45), and the rostral inferior parietal cortex BA40 (merged PFop, PFt, PF, PFm, and PFcm) were spatially defined using probabilistic cytoarchitectonic maps using the SPM anatomy toolbox v.1.6. To delineate auditory regions, including Heschl’s gyrus (BA41/BA42), the middle temporal gyrus (BA21) and the caudal inferior parietal cortex (BA39), we used the aal atlas implemented in xjview based on a macroscopic anatomical parcellation of the MNI MRI Single-Subject Brain^6^.

Pearson’s correlations across the nine regions were computed between rest and movie conditions, from the EEG-BOLD partial correlation values (1–70 Hz) obtained for each region and subject [see fMRI-Informed EEG (Partial Correlations)]. We obtained two matrices (one per group) consisting of one correlation value per region and subject, reflecting the spectrum similarity between conditions. Statistical significance of the correlation values of each matrix was tested using one-sample *t*-tests. The resulting two matrices of significant (positive and negative) correlations were then compared between groups using two-tailed two-sample *t*-tests (**Figure 4C**). We previously argued (Morillon et al., 2010) that such a matrix may be interpreted in a directional way, under the double assumption that (i) the oscillatory profile observed in a given region at rest determines the oscillatory profile observed in regions that receive its input during the movie, and (ii) the resting profile in one region cannot be explained out by the movie profile in another region of the same functional network. Significant differences between groups are represented in **Figure 4D**. This matrix can be interpreted in a directional way, as we hypothesize that the resting state profile determines lateralization of the language network during the movie. Arrows pointing from one brain region A to another brain region B indicate significant differences between the EEG-BOLD spectrum at rest in area A and the pattern in area B during movie viewing between groups. Note that in **Figure 4** the different territories corresponding to one functional area were pooled together to facilitate visualization (i.e., BA4, Broca). All statistical analyses were performed using Matlab v11b (The MathWorks Inc., Natick, MA, USA).

## Results

**FIGURE 1:**
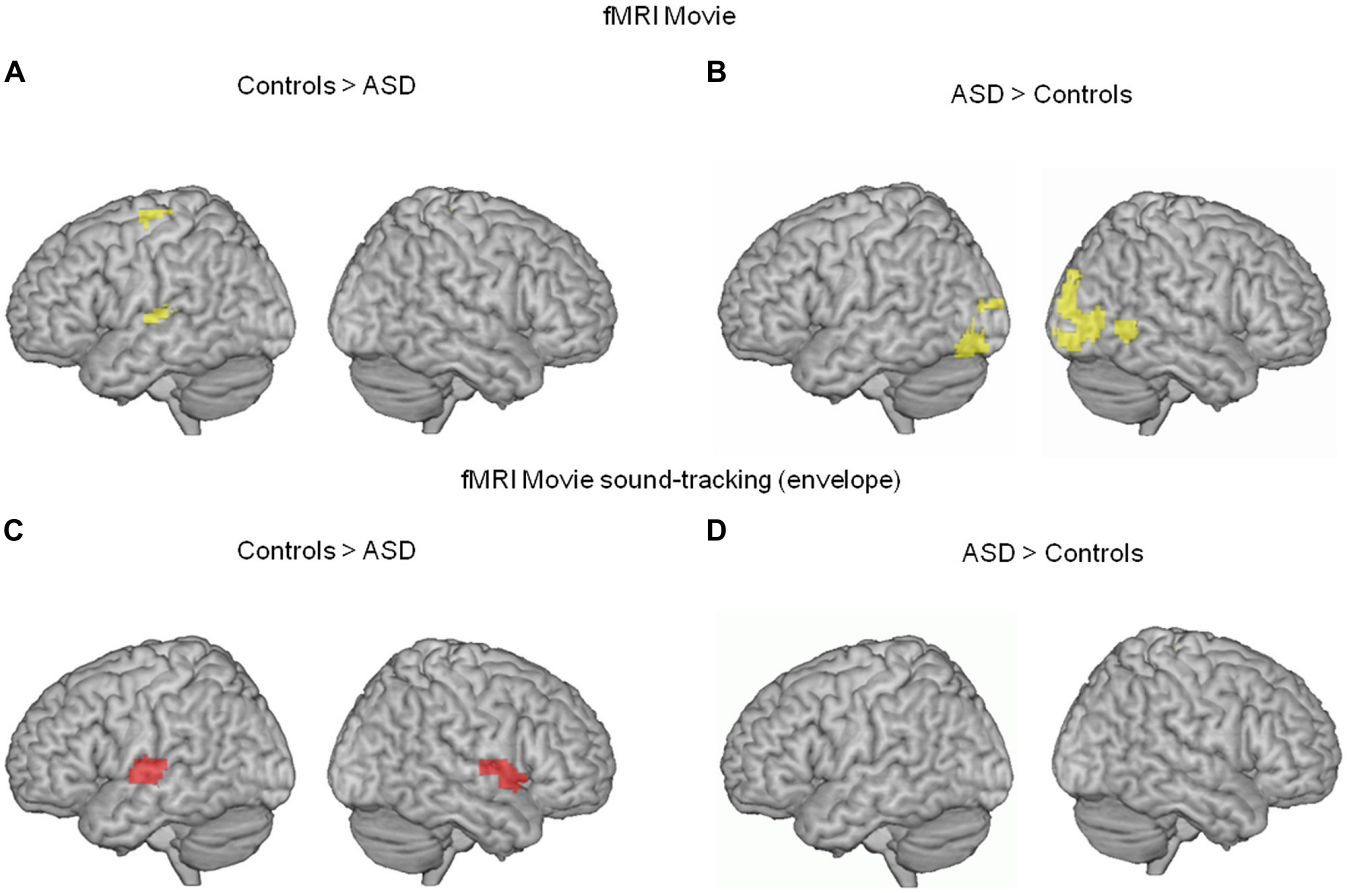
**(A,B)** Comparison of neural activity blood oxygen-level dependent (BOLD) in 13 subjects with autism and 13 unaffected controls, when they were watching a scientific TV program (vs. rest). In autism, neural activity was reduced in the left supplementary motor area and left auditory cortex (whole brain interaction *p* < 0.02, uncorrected; Heschl’s gyrus, *p* = 0.02 FWE corrected; 54, -19, 7 MNI coordinates), and enhanced in bilateral visual cortex (whole brain *p* < 0.01 uncorrected, 3,-91,-8 MNI coordinates). **(C,D)** To further specify the effect, we compared the BOLD response to the movie soundtrack envelope, i.e., the syllabic envelope of speakers’ speech, between the two groups. In ASD, the left auditory cortex showed reduced syllable tracking (-63, -13, 7 MNI coordinates, left Heschl’s gyrus *p* = 0.016 FWE corrected; *p* = 0.156 uncorrected in right Heschl’s gyrus, 62, -22, 7 MNI coordinates).

We first analyzed the fMRI data using a simple contrast of movie vs. rest in each group. BOLD responses to the movie occurred in visual and auditory brain areas in both groups, yet were less pronounced in the ASD group in left superior parietal and superior temporal cortices (auditory cortex, **Figure 1A**). Conversely, movie-related BOLD activity was enhanced in autism relative to controls in bilateral non-primary visual cortex and the right posterior superior temporal sulcus (**Figure 1B**).

To more precisely characterize the reduced auditory cortical response in ASD, we computed a regressor from the temporal envelope of the movie soundtrack. This regressor primarily indexes syllable boundaries in the speakers’ discourse (Ghitza, 2012). Critically, because there was continuous speech throughout the movie with an alternation between off-voices and speakers facing the audience, the regressor was specific to speech and controlled for concurrent visual processing of faces. ASD participants showed a deficit in speech envelope tracking, as assessed by the BOLD signal, in a region of auditory cortex that overlapped with the region showing a global response deficit to the movie (**Figures 1C,D**). These initial two analyses of the fMRI data alone indicate deficient auditory processing in ASD, and show that this deficit is related to atypical speech tracking at the syllabic timescale.

A quantitative reduction in speech tracking as observed in the fMRI data could be a consequence of the failure of slow speech modulations to engage theta-range activity in auditory cortex during speech stimulation (Ghitza, 2012; Peelle et al., 2013). We therefore next addressed whether in ASD EEG anomalies in the theta range were associated with the inability of auditory cortex to optimally represent the soundtrack envelope. The simultaneous EEG and fMRI recordings allowed us to explore how theta power fluctuations driven by the movie correlate with local synaptic activity in auditory cortex, as indexed by the BOLD signal (Magri et al., 2012; see Materials and Methods). In both groups, theta-BOLD coupling localized to bilateral superior temporal gyri (**Figure S1**).

**FIGURE 2:**
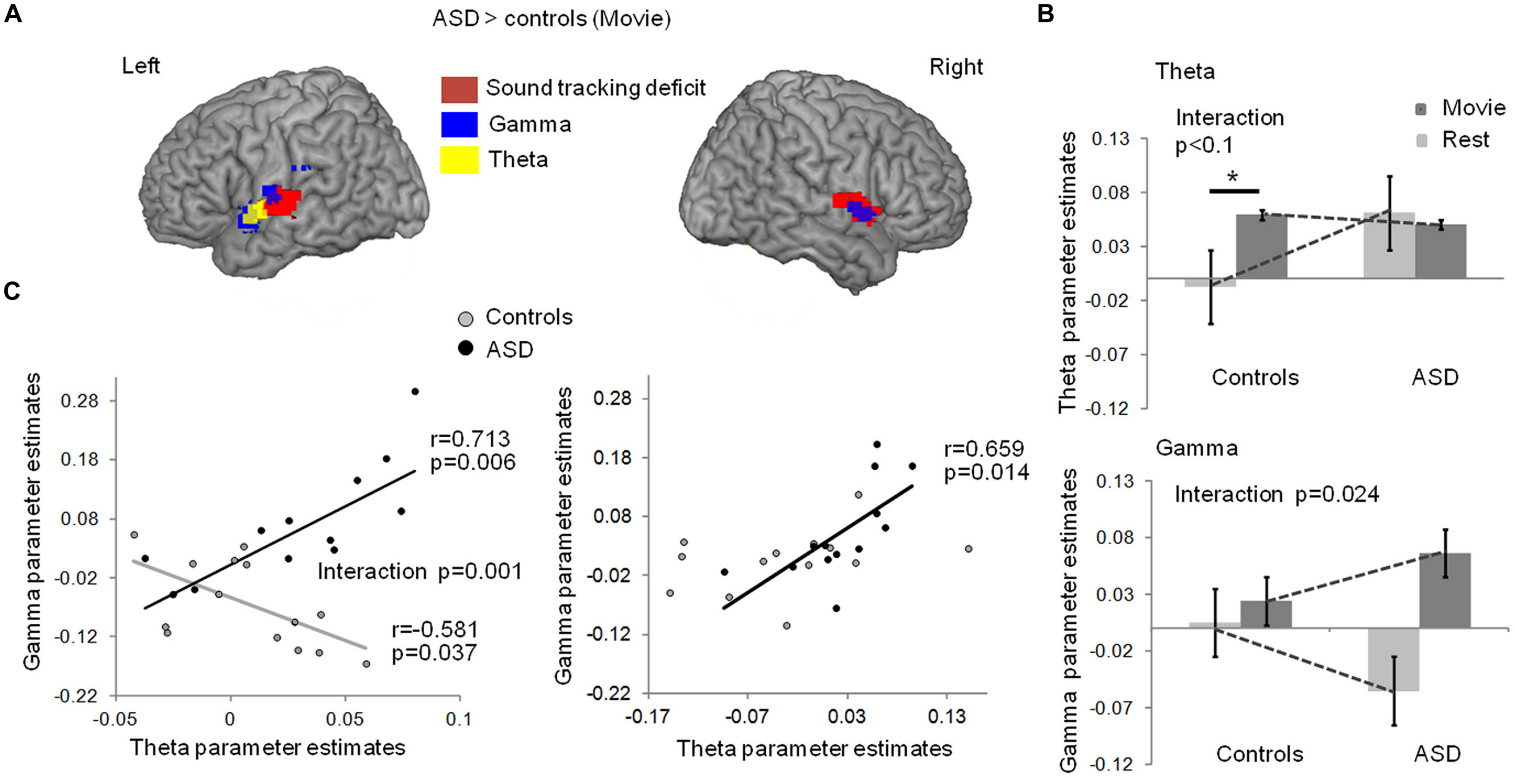
**(A)** Comparison of EEG-BOLD coupling between groups with and without autism, in theta and low-gamma bands, during movie viewing. Subjects with autism had enhanced theta-BOLD (blue, whole brain *p* < 0.01 uncorrected, -48, -1, -5 MNI coordinates; left Heschl’s gyrus, *p* = 0.034 FWE) and gamma-BOLD (green, left panel, *p* < 0.01, -54, -7, 10 MNI coordinates; left Heschl’s gyrus *p* = 0.007 FWE) coupling in the left superior temporal lobe relative to controls; subjects with ASD had enhanced gamma-BOLD coupling (green, right panel, *p* < 0.05, 51, -1, 1 MNI coordinates) in the right temporal lobe relative to controls. **(B)** EEG-BOLD coupling at rest and during movie viewing in each group, within the theta (up panel) and gamma (bottom panel) frequency bands. The regions were sampled from the left auditory cortex, at the location where there was a significant theta EEG-BOLD effect during the movie in controls (up panel), and a significant decrease in gamma-BOLD coupling at rest in the ASD group (bottom panel). **(C)** Left panel: in controls, gamma- and theta-BOLD coupling in left auditory cortex were negatively related, in line with a control of gamma by theta activity. In autism, an inverted relation suggests atypical theta/gamma interaction. The group interaction was significant at *p* = 0.001; Right panel: in the right temporal lobe, the anomaly in autism was less pronounced and the negative correlation between theta and gamma was not present in controls (*p* = 0.243). * indicates a significant difference with *p* < 0.05.

Stronger theta-BOLD coupling in young adults with autism relative to controls was detected during the movie in left Heschl’s gyrus [*p* = 0.03, familywise error (FWE) corrected in Heschl’s gyrus] at the anterior border of the auditory cortex (**Figure 2A**, blue). This effect spatially overlapped with the envelope-tracking deficit as defined using fMRI responses to the movie (**Figures 1C** and **2A**). We then went on to compare theta EEG-BOLD coupling at rest and during the movie, in the auditory cortex region where there was a significant theta EEG-BOLD effect in controls during the movie (anterior to auditory cortex). In this region subjects with autism had enhanced resting theta-BOLD coupling relative to controls, and theta-BOLD coupling did not increase when they were exposed to speech (**Figure 2B**, top panel). In sum, unlike in controls, theta activity was already present in auditory cortex at rest and did not increase with speech stimulation. Note, however, that we observed a non-significant theta-BOLD coupling increase at 8 Hz in subjects with ASD during the movie. This small effect was hence outside the typical 4–7 Hz theta range. Taken together, our data indicate that subjects with autism have abnormal theta responses to speech. As it has been established that speech intelligibility depends on the strength of theta phase-locking to the most prominent modulations in speech (Peelle et al., 2013) that typically occurs at 4 Hz, atypical theta engagement in response to speech could be one key contributing factor to explain anomalies of language processing in autism (Eyler et al., 2012).

Theta activity has been argued to be important in speech decoding (Luo and Poeppel, 2007; Henry and Obleser, 2012) because, among other reasons, it orchestrates gamma activity and the timing of cortical population spiking (Kayser et al., 2012). Mechanistically, this orchestration might serve to package information in time frames that can be read out and decoded at the next hierarchical stage (Shamir et al., 2009; Ghitza, 2011). We therefore, in a next step, addressed the distribution of gamma power/BOLD correlations throughout the brain during the movie (**Figure S1**). We found that gamma power/BOLD correlations were enhanced in subjects with autism relative to controls in bilateral auditory cortices, in particular in the left auditory cortex, at its junction with the supramarginal region in the upper bank of the Sylvian fissure and the insula (**Figure 2A**, green). In these regions, the group difference was significant at *p* = 0.007 (FWE corrected). This effect overlapped with the region where (i) BOLD activity was reduced in ASD during the movie (**Figure 1A**), (ii) speech envelope tracking by fMRI responses was deficient (**Figure 1B** and **2A**), and (iii) theta-correlated BOLD signal was atypical (**Figure 2A**). Controls displayed a weak gamma-BOLD coupling at rest that only moderately increased during the movie. This suggests that the movie induced a temporal reorganization of gamma activity, presumably via theta activity, rather than strong power variations (Benchenane et al., 2010; Kayser et al., 2012). By contrast, subjects with autism showed a marked negative gamma-BOLD coupling at rest and a stronger than normal positive gamma-BOLD coupling during the movie (**Figure 2B**, bottom panel, group × condition interaction *p* = 0.024), confirming abnormal gamma generation (Edgar et al., 2013) and reactivity to sound modulations.

To ascertain the specificity of these effects for the theta and gamma bands, we explored EEG-BOLD coupling across the full recorded EEG spectrum, focusing principally on the left auditory region that was more activated in control than ASD subjects during movie viewing. We observed significantly enhanced EEG-BOLD coupling in autism during movie viewing between 25 and 35 Hz (**Figure S2**, left panel), i.e., in a range previously related to phonemic processing by auditory cortex (Lehongre et al., 2011). As an additional control for the auditory specificity of theta and gamma effects, we computed correlations between whole spectrum EEG and BOLD signal in the left visual region that was over-activated during the movie (fusiform gyrus, **Figure 1**). In this occipital region, we observed a non-significant reduction in gamma-BOLD correlations in ASD relative to controls (**Figure S2**, right panel). This control offers qualitative support to recent studies showing that gamma activity is reduced in ASD relative to controls in response to faces (Khan et al., 2013). Importantly, such data show that synaptic activity as indexed by the BOLD signal does not systematically translate into strong oscillatory effects (Logothetis, 2010), such as those we observe in the left auditory cortex.

Critically, as our speech processing model assumes that the modulation of gamma activity by theta activity is essential to speech comprehension (Giraud and Poeppel, 2012), we explored how theta and gamma power fluctuations covaried during movie viewing, in Heschl’s gyrus. Because scanner noise and motion artifacts more strongly affected phase than power signals, we could not directly assess theta/gamma phase-amplitude coupling. Instead we approximated theta-gamma power relationship by regressing the gamma-BOLD parameter estimates onto the theta-BOLD ones. We observed a negative relationship in controls in left auditory cortex [*r*(13) = -0.58, *p* = 0.037, **Figure 2C**, left], confirming a functional dependency between theta and gamma under physiological conditions, compatible with gamma activity being down-regulated by theta activity. In autism this dependency was reversed [*r*(13) = 0.7; *p* = 0.006, group × frequency-range interaction significant at *F*(1,22) = 15.767; *p* = 0.001], suggesting atypical coordination between gamma and theta activity, presumably in relation to an absence of down-regulation. The group interaction was not significant in the right temporal cortex [*F*(1,22) = 0.872; *p* = 0.361] where controls showed no theta-gamma dependency (**Figure 2C**, right), in line with the specificity of left auditory cortex for speech processing (Gross et al., 2013).

**FIGURE 3:**
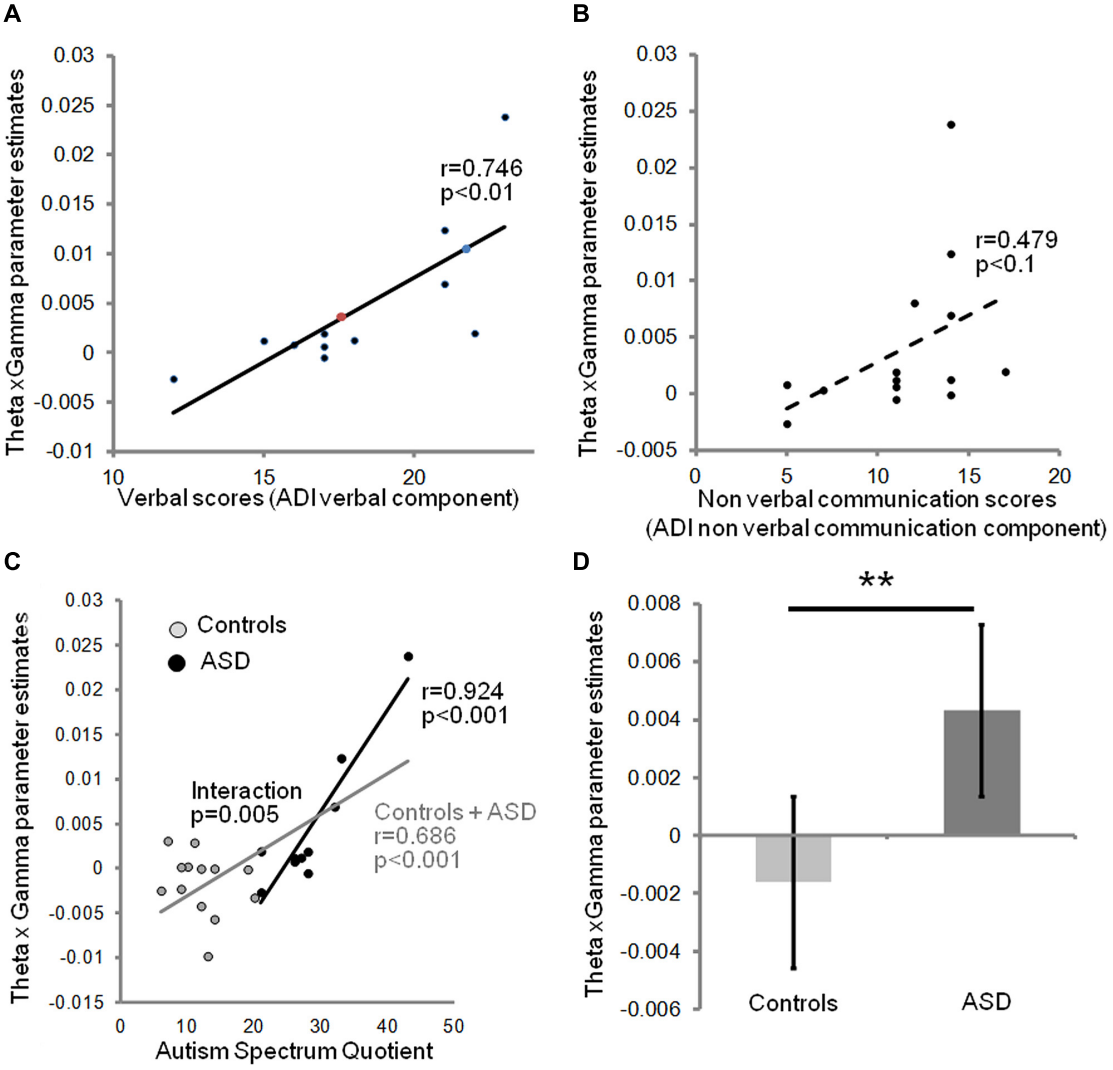
Relationship between theta/gamma-BOLD interaction term, and clinical data: the neurophysiological theta × gamma variable predicts the verbal component of ADI **(A)**, but not the non-verbal communication component **(B)**. Colored dots indicate the predicted ADI score of the dysphasic subjects: the blue one was deeply dysphasic and the red one was moderately dysphasic; note the high ADI predicted score of the non-verbal one. The theta × gamma variable closely predicts autism severity assessed by autism spectrum quotients (AQs) in the affected group, and autism traits across groups, but not within the control group **(C)**. There is a significant group interaction (*p* = 0.005) confirming a non-linear shift in the dependency between autism traits and auditory theta/gamma coupling when moving to AQ scores associated with ASD. **(D)** Theta × gamma parameter significantly distinguishes the two groups (Spearman *t*-test, *p* = 0.01). Error bars represent SEM. ** indicates a significant difference with *p* < 0.01.

We next investigated the relation between the severity of autism clinical symptoms and the observed anomalies of oscillatory responses to speech in auditory cortex. We constructed a neurophysiological variable that combined theta and gamma activity. Because theta and gamma variables were not independent, we excluded a linear combination of gamma and theta parameter estimates (theta + gamma + theta × gamma), but correlated the behavioral data with the interaction term (theta × gamma), which is sensitive to the sign of the correlation. This composite variable predicted subjects’ verbal scores on the ADI test [*r*(11) = 0.746; *p* = 0.008], but only weakly correlated with non-verbal scores in the ASD group (**Figures 3A,B**). This observation is consistent with the view that the absence of a canonical theta-gamma dependency is specifically related to language difficulties. Note that no such effects were present in right auditory cortex (group × hemisphere interaction *p* < 0.001). Interestingly, the theta × gamma variable also predicted the AQ across groups [*r*(23) = 0.68, *p* = 0.000, **Figure 3C**], reflecting the large and significant group difference in theta/gamma coupling (**Figure 3D**). Most importantly, the neurophysiological index of theta-gamma dependency was strongly tied to the autism symptoms; within the ASD group the correlation attained *r*(10) = 0.924; *p* = 0.000, with a group interaction of *F*(1,19) = 10.135; *p* = 0.005.

**FIGURE 4:**
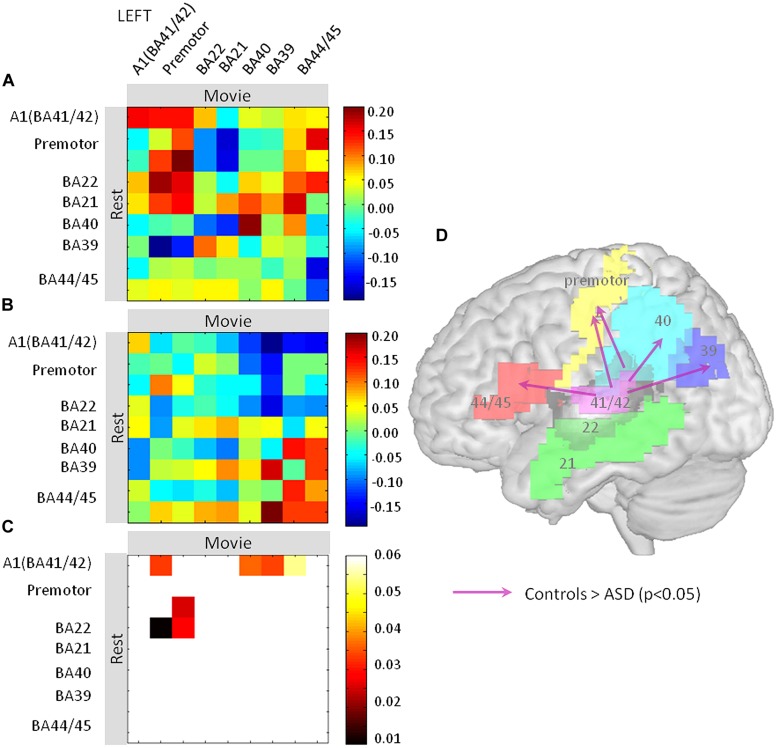
Oscillation-based functional disconnection of auditory cortex in autism. **(A,B)** We quantified how the oscillatory spectrum in auditory cortex at rest propagated to the other regions of the left lateralized language network during the movie, in controls **(A)** and in ASD subjects **(B)**. We used Pearson’s correlation matrices of the EEG-BOLD correlations over the whole spectrum (1–70 Hz) across nine regions of the language system. The results are directional under the non-reciprocal assumption that the resting oscillatory profile is a determinant of the oscillatory profile during the movie. **(C)** Similarity matrices were compared between groups and statistics are reported at *p* < 0.05. **(D)** Statistics are reported on a brain template; they show that the primary auditory cortex (BA41/42) has reduced connectivity with Brodmann Areas 44, 45, 40, 39, and the premotor cortex relative to controls.

Finally, we assessed how the oscillatory spectral profiles and theta-gamma relationship observed at rest in left auditory cortex related to effects in the distributed language network during the movie. We computed EEG (1–70 Hz)-BOLD coupling from nine language regions of the left hemisphere during rest and movie viewing and correlated it across the two conditions (**Figure 4**). We interpret these findings as directional oscillation-based connectivity, under the hypothesis that EEG-BOLD coupling at rest predicts EEG-BOLD coupling during movie viewing (see Materials and Methods and Morillon et al., 2010). The notion of connectivity is based on the capacity of one region to inherit, during the movie part of the experiment, the oscillatory profile observed at rest in another region. Using this approach, we observed that left auditory cortex was more weakly coupled to Broca’s area (BA 44/45), BA39 and 40, and the premotor cortex in ASD than in controls. This pattern suggests that the propagation of the broad-spectrum oscillatory profile in auditory cortex to key regions of the language network was reduced in subjects with autism relative to controls (**Figure 4**). Importantly, there was reduced connectivity from A1 (BA41/BA42) to Broca’s area and motor cortex, but not from Broca’s area and motor cortex to A1, indicating that the anomaly is likely primarily auditory. Because the oscillatory profile determines the time constants with which speech is segmented, and the neural code presented to higher order language brain regions due to temporally spike reorganization, functional isolation of auditory cortex should strongly impair on-line speech decoding.

## Discussion

The current findings show severe anomalies of auditory cortical activity at rest and in response to speech in subjects with ASD, affecting conjointly the theta and the low-gamma frequency bands. Cortical oscillations arise from excitatory–inhibitory interactions within and across specific cortical laminae (Cannon et al., 2014), and auditory oscillation anomalies represent a plausible functional counterpart to the structural disorganization of language cortices and the disruption of cortical inhibition previously shown in ASD (Rojas et al., 2013). When subjects with ASD engaged in natural activities that do not place specific emphasis on social functions, their left speech processing regions manifested a primary deficit. In ASD, the auditory cortex reacted less to speech syllabic modulations, which were also weakly tracked by theta oscillations. Such an anomaly could have severe functional consequences on speech perception, since disrupted theta tracking of speech modulations results in less efficient syllable encoding and reduced intelligibility (Ahissar et al., 2001; Henry and Obleser, 2012; Peelle et al., 2013; Doelling et al., 2014). From a theoretical perspective, intelligibility difficulties could occur because atypical theta tracking compromises syllable parsing, a process by which theta oscillations locked on syllable onsets determine syllable-based windows of integration, and temporally organize the neural activity that is passed to higher hierarchical processing levels (Ghitza, 2011). The current results are consistent with recent findings showing enlarged temporal windows of integration in audio-visual speech in autism (Stevenson et al., 2014b). In control subjects, there is a 250–300 ms tolerance to audio and visual asynchrony in speech, suggesting that visual and sound tracks could be integrated via a theta-based mechanism (Luo et al., 2010). In subjects with autism, the sensitivity to audio-visual speech asynchrony is dramatically blurred, with temporal windows of integration reaching up to 1 s (Stevenson et al., 2014b). These observations converge with ours to suggest a severe disruption of theta-based speech integration mechanisms in autism.

Our data further show that speech-driven theta and gamma neural oscillations lack the typical physiological coordination. Unlike in controls, there was no sign of down-regulation of gamma by theta activity during speech processing in ASD, but rather an opposite dependency such that gamma and theta-BOLD coupling jointly increased out of physiological ranges. According to recent oscillation-based models of speech processing (Giraud and Poeppel, 2012), dysfunctional theta/gamma coordination should disrupt the alignment of neuronal excitability with syllabic onset, and compromise speech decoding. The observation that theta/gamma balance was not merely disrupted in ASD but reversed relative to controls could signal pathological wiring patterns within/across cortical microcircuits in autism.

We also found that the atypical interaction between theta and gamma responses to speech strongly correlated with clinical variables. The oscillatory anomalies matched not only the verbal impairment, but more broadly the severity of autism symptoms. These findings underscore the central place of sensory anomalies in ASD (Marco et al., 2011) and open up a possibility to consider sensory disturbances in relation to the complex spectrum of cognitive symptoms. By reducing the ability to temporally organize speech information, altered coordinated neural activity in auditory cortex and disrupted oscillation-based connectivity with Broca’s area and motor cortex likely compromise the ability of ASD subjects to respond appropriately to speech signals and to interact with their peers. As illustrated by the increased prevalence of the autism phenotype in children with profound hearing loss (Snowling et al., 2003), auditory-based communication appears of crucial importance for normal cognitive development, and dysfunctional auditory processing could contribute to the social isolation of subjects with autism.

On the other hand, dysfunctional speech-related neural processing in the autistic brain might also denote a deficiency of oscillation coordination, based on temporal integration deficits, that reaches beyond the auditory modality. Given the broad spectrum of sensory and cognitive symptoms in autism, anomalies of oscillatory entrainment and coupling may be more pervasive than currently appreciated. Adjudicating between a primary auditory deficit and a generic deficit of oscillatory function in autism (Dinstein et al., 2011) would require in-depth investigations of oscillatory brain responses in other functional domains besides speech processing. In our present study, some preliminary evidence in favor of a primary impairment of auditory integration may come from the observation that abnormal synaptic activity levels (fMRI) and oscillation anomalies co-occurred in auditory cortex, but were dissociated in visual regions. This observation could indicate a compensatory role of visual processing in autism during speech perception, as supported by the observation that subjects with ASD extensively explore the mouth region in face-to-face situations (Klin et al., 2002), and use specific attention modes to enhanced local visual processing (Schwarzkopf et al., 2014).

Although the current findings point to a primary dysfunction of oscillatory activity resulting in a speech-tracking deficit, this study, unsurprisingly, has some important limitations. First, given that we had to select subjects who could stay confined in an MRI scanner with the EEG equipment, our ASD sample is relatively small, which necessarily limits the generalizability of the findings. A second limitation might be seen in the fact that the two groups of subjects were not matched for IQ as is usually the case in cognitive studies of autism. However, because our hypotheses focused on low-level automatic auditory tracking by auditory cortex, we chose to include all levels of autism, and IQs, only excluding subjects with Asperger syndrome. Our aim here was to work with a sample representative of the diversity of the autism population, which implies IQ and speech proficiency differences with the control group. However, by correcting the statistics for IQ we finally report group effects that are not primarily explained by this factor. That the results were confined to auditory cortices indicate that this strategy was useful. The broad spectrum of autism severity used here also provided a good sensitivity in correlation analyses, and even the dysphasic ASD subjects were not detected as outliers (**Figures 2** and **3**). A third potential confound when comparing groups lies in head motion, which is invoked as a strong bias in neuroimaging findings (Pelphrey and Deen, 2012). Thanks to EEG recordings, we most likely circumvented this potentially serious issue by excluding subjects and recording periods showing motion artifacts, and we further corrected for head motion parameters in the statistics after verifying that there was no residual outlier for motion. Combining EEG with fMRI makes it unlikely that artifacts of dual origin are reflected in a differential activity precisely in auditory cortices. Finally, although we used EEG to check that subjects were not asleep during the experiment, we cannot ensure that subjects with and without autism maintained comparable levels of auditory attention. There is even reason to believe that – if our hypothesis that subjects with autism have reduced ability to follow speech signals due to oscillatory dysfunction is correct – there should be detrimental consequences for implementing auditory attentional control. It has been shown that auditory attention acts by phase resetting slow oscillations (Kayser, 2009; Zion Golumbic et al., 2013), which in turn enhances the control of gamma by theta oscillations (Sauseng et al., 2008). From a neurophysiological perspective, one can therefore expect speech tracking and attentional mechanisms to be inherently intertwined.

The current findings support a model that relates the coordination of cortical oscillations to temporal integration of the sensory input. The data could be useful for understanding the exact pathogenetic mechanisms of abnormal sensory reactivity in autism. Whether restricted to the auditory modality or more widespread, the lack of coordination across slow (theta) and fast (gamma) oscillations suggests a deficit in information integration at two timescales that could also have important consequences on the ability to manipulate mental representations of different orders, here phonemes and syllables. The present study should be considered as a first attempt at understanding whether speech related oscillatory activity was impaired in autism, and is expected to be followed by others that should clarify the relationship between phenotype and neurophysiology, using detailed evaluation of linguistic skills. Finally, speech reception disturbances in ASD could constitute an interesting possible entry point to clinical handling, as oscillatory activity can be focally modulated, e.g., by neuro-feedback or non-invasive transcranial stimulation (Engelhard et al., 2013).

## Footnotes

1 www.fil.ion.ucl.ac.uk

2 http://wiki.cenir.org/doku.php/datahandler

3 www.montefiore.ulg.ac.be/∼phillips/FASST.html

4 http://sccn.ucsd.edu/eeglab

5 http://cibsr.stanford.edu/tools/human-brain-project/artrepair-software.html

6 www.alivelearn.net/xjview8/

7 http://www.sph.sc.edu/comd/rorden/mricron

8 http://fieldtrip.fcdonders.nl/

## Supplementary material

Figure S1 **Whole brain topography of theta-BOLD (top) and low-gamma-BOLD (bottom) effects in controls (left) and subjects with autism during the movie (whole brain, *p* < 0.01 uncorrected; whole brain, *p* < 0.005 uncorrected; left Heschl’s gyrus *p* < 0.05 FWE corrected)**.

Figure S2 **(A)** Neural activity (fMRI only, top panels) in subjects with autism (right) and controls (left) during audio-visual presentation of the documentary (*p* < 0.05, corrected). **(B)** Partial correlations between the EEG power spectrum (1–70 Hz) and fMRI data at the left hemispheric locations where we found reduced (auditory cortex) and enhanced (visual cortex) neural activity. Note that, unlike the effect in left auditory cortex, the effect in the right posterior superior temporal sulcus is not explained by differences in EEG-BOLD correlations.

## References

[B1] Abrams D. A. Lynch C. J. Cheng K. M. Phillips J. Supekar K. Ryali S. (2013). Underconnectivity between voice-selective cortex and reward circuitry in children with autism. *Proc. Natl. Acad. Sci. U.S.A.* 110 12060–12065 10.1073/pnas.1302982110 23776244 PMC3718181

[B2] Ahissar E. Nagarajan S. Ahissar M. Protopapas A. Mahncke H. Merzenich M. M. (2001). Speech comprehension is correlated with temporal response patterns recorded from auditory cortex. *Proc. Natl. Acad. Sci. U.S.A.* 98 13367–13372 10.1073/pnas.201400998 11698688 PMC60877

[B3] Ainsworth M. Lee S. Cunningham M. O. Roopun A. K. Traub R. D. Kopell N. J. (2011). Dual γ rhythm generators control interlaminar synchrony in auditory cortex. *J. Neurosci.* 31 17040–17051 10.1523/JNEUROSCI.2209-11.2011 22114273 PMC3654396

[B4] American Psychiatric Association [APA]. (2013). *Diagnostic** and Statistical Manual of Mental Disorders*, 5th Edn. (Arlington: American Psychiatric Association).

[B5] Amiez C. Petrides M. (2009). Anatomical organization of the eye fields in the human and non-human primate frontal cortex. *Prog. Neurobiol.* 89 220–230 10.1016/j.pneurobio.2009.07.010 19665515

[B6] Baron-Cohen S. Wheelwright S. Skinner R. Martin J. Clubley E. (2001). The autism-spectrum quotient (AQ): evidence from Asperger syndrome/high-functioning autism, males and females, scientists and mathematicians. *J. Autism Dev. Disord.* 31 5–17 10.1023/A:1005653411471 11439754

[B7] Bartos M. Vida I. Jonas P. (2007). Synaptic mechanisms of synchronized gamma oscillations in inhibitory interneuron networks. *Nat. Rev. Neurosci.* 8 45–56 10.1038/nrn2044 17180162

[B8] Benchenane K. Peyrache A. Khamassi M. Tierney P. L. Gioanni Y. Battaglia F. P. (2010). Coherent theta oscillations and reorganization of spike timing in the hippocampal- prefrontal network upon learning. *Neuron* 66 921–936 10.1016/j.neuron.2010.05.013 20620877

[B9] Buzsáki G. Anastassiou C. A. Koch C. (2012). The origin of extracellular fields and currents–EEG, ECoG, LFP and spikes. *Nat. Rev. Neurosci.* 13 407–420 10.1038/nrn3241 22595786 PMC4907333

[B10] Cannon J. McCarthy M. M. Lee S. Lee J. Börgers C. Whittington M. A. (2014). Neurosystems: brain rhythms and cognitive processing. *Eur. J. Neurosci.* 39 705–719 10.1111/ejn.12453 24329933 PMC4916881

[B11] Chase A. (2014). Brain imaging: white matter disruption in autism spectrum disorder is exaggerated by head movements during neuroimaging. *Nat. Rev. Neurol.* 10 122 10.1038/nrneurol.2014.20 24514871

[B12] Courchesne E. Carper R. Akshoomoff N. (2003). Evidence of brain overgrowth in the first year of life in autism. *JAMA* 290 337–344 10.1001/jama.290.3.337 12865374

[B13] Ding N. Simon J. Z. (2013). Robust cortical encoding of slow temporal modulations of speech. *Adv. Exp. Med. Biol.* 787 373–381 10.1007/978-1-4614-1590-9 23716243 PMC13297007

[B14] Dinstein I. Heeger D. J. Lorenzi L. Minshew N. J. Malach R. Behrmann M. (2012). Unreliable evoked responses in autism. *Neuron* 75 981–991 10.1016/j.neuron.2012.07.026 22998867 PMC3457023

[B15] Dinstein I. Pierce K. Eyler L. Solso S. Malach R. Behrmann M. (2011). Disrupted neural synchronization in toddlers with autism. *Neuron* 70 1218–1225 10.1016/j.neuron.2011.04.018 21689606 PMC3119852

[B16] Doelling K. B. Arnal L. H. Ghitza O. Poeppel D. (2014). Acoustic landmarks drive delta-theta oscillations to enable speech comprehension by facilitating perceptual parsing. *Neuroimage* 85(Pt 2), 761–768 10.1016/j.neuroimage.2013.06.035 23791839 PMC3839250

[B17] Edgar J. C. Khan S. Y. Blaskey L. Chow V. Y. Rey M. Gaetz W. (2013). Neuromagnetic oscillations predict evoked-response latency delays and core language deficits in autism spectrum disorders. *J. Autism Dev. Disord.* 45 395–405 10.1007/s10803-013-1904-x 23963591 PMC5012005

[B18] Engelhard B. Ozeri N. Israel Z. Bergman H. Vaadia E. (2013). Inducing γ oscillations and precise spike synchrony by operant conditioning via brain-machine interface. *Neuron* 77 361–375 10.1016/j.neuron.2012.11.015 23352171

[B19] Eyler L. T. Pierce K. Courchesne E. (2012). A failure of left temporal cortex to specialize for language is an early emerging and fundamental property of autism. *Brain* 135 949–960 10.1093/brain/awr364 22350062 PMC3286331

[B20] Fries P. (2009). The model- and the data-gamma. *Neuron* 64 601–602 10.1016/j.neuron.2009.11.024 20005817

[B21] Friston K. (2012). Ten ironic rules for non-statistical reviewers. *Neuroimage* 61 1300–1310 10.1016/j.neuroimage.2012.04.018 22521475

[B22] Gervais H. Belin P. Boddaert N. Leboyer M. Coez A. Sfaello I. (2004). Abnormal cortical voice processing in autism. *Nat. Neurosci.* 7 801–802 10.1038/nn1291 15258587

[B23] Ghitza O. (2011). Linking speech perception and neurophysiology: speech decoding guided by cascaded oscillators locked to the input rhythm. *Front. Psychol.* 2:130 10.3389/fpsyg.2011.00130 PMC3127251 21743809

[B24] Ghitza O. (2012). On the role of theta-driven syllabic parsing in decoding speech: intelligibility of speech with a manipulated modulation spectrum. *Front. Psychol.* 3:238 10.3389/fpsyg.2012.00238 PMC3397378 22811672

[B25] Ghitza O. Greenberg S. (2009). On the possible role of brain rhythms in speech perception: intelligibility of time-compressed speech with periodic and aperiodic insertions of silence. *Phonetica* 66 113–126 10.1159/000208934 19390234

[B26] Giraud A.-L. Kleinschmidt A. Poeppel D. Lund T. E. Frackowiak R. S. J. Laufs H. (2007). Endogenous cortical rhythms determine cerebral specialization for speech perception and production. *Neuron* 56 1127–1134 10.1016/j.neuron.2007.09.038 18093532

[B27] Giraud A.-L. Poeppel D. (2012). Cortical oscillations and speech processing: emerging computational principles and operations. *Nat. Neurosci.* 15 511–517 10.1038/nn.3063 22426255 PMC4461038

[B28] Gross J. Hoogenboom N. Thut G. Schyns P. Panzeri S. Belin P. (2013). Speech rhythms and multiplexed oscillatory sensory coding in the human brain. *PLoS Biol.* 11:e1001752 10.1371/journal.pbio.1001752 PMC3876971 24391472

[B29] Henry M. J. Obleser J. (2012). Frequency modulation entrains slow neural oscillations and optimizes human listening behavior. *Proc. Natl. Acad. Sci. U.S.A.* 109 20095–20100 10.1073/pnas.1213390109 23151506 PMC3523826

[B30] Jacot-Descombes S. Uppal N. Wicinski B. Santos M. Schmeidler J. Giannakopoulos P. (2012). Decreased pyramidal neuron size in Brodmann areas 44 and 45 in patients with autism. *Acta Neuropathol.* 124 67–79 10.1007/s00401-012-0976-6 22467063

[B31] Kayser C. (2009). Phase resetting as a mechanism for supramodal attentional control. *Neuron* 64 300–302 10.1016/j.neuron.2009.10.022 19914178

[B32] Kayser C. Ince R. A. A. Panzeri S. (2012). Analysis of slow (theta) oscillations as a potential temporal reference frame for information coding in sensory cortices. *PLoS Comput. Biol.* 8:e1002717 10.1371/journal.pcbi.1002717 PMC3469413 23071429

[B33] Khan S. Gramfort A. Shetty N. R. Kitzbichler M. G. Ganesan S. Moran J. M. (2013). Local and long-range functional connectivity is reduced in concert in autism spectrum disorders. *Proc. Natl. Acad. Sci. U.S.A.* 110 3107–3112 10.1073/pnas.1214533110 23319621 PMC3581984

[B34] Klin A. Jones W. Schultz R. Volkmar F. Cohen D. (2002). Visual fixation patterns during viewing of naturalistic social situations as predictors of social competence in individuals with autism. *Arch. Gen. Psychiatry* 59 809–816 10.1001/archpsyc.59.9.809 12215080

[B35] Kujala T. Lepistö T. Näätänen R. (2013). The neural basis of aberrant speech and audition in autism spectrum disorders. *Neurosci. Biobehav. Rev.* 37 697–704 10.1016/j.neubiorev.2013.01.006 23313648

[B36] Laufs H. Holt J. L. Elfont R. Krams M. Paul J. S. Krakow K. (2006). Where the BOLD signal goes when alpha EEG leaves. *Neuroimage* 31 1408–1418 10.1016/j.neuroimage.2006.02.002 16537111

[B37] Lehongre K. Ramus F. Villiermet N. Schwartz D. Giraud A.-L. (2011). Altered low-γ sampling in auditory cortex accounts for the three main facets of dyslexia. *Neuron* 72 1080–1090 10.1016/j.neuron.2011.11.002 22196341

[B38] Logothetis N. K. (2010). Bold claims for optogenetics. *Nature* 468 E3–E4 10.1038/nature09532 21107378

[B39] Lord C. Rutter M. Le Couteur A. (1994). Autism Diagnostic Interview-Revised: a revised version of a diagnostic interview for caregivers of individuals with possible pervasive developmental disorders 1. 24. *J. Autism Dev. Disord*. 24 659–685 10.1007/BF02172145 7814313

[B40] Luo H. Liu Z. Poeppel D. (2010). Auditory cortex tracks both auditory and visual stimulus dynamics using low-frequency neuronal phase modulation. *PLoS Biol.* 8:e1000445 10.1371/journal.pbio.1000445 PMC2919416 20711473

[B41] Luo H. Poeppel D. (2007). Phase patterns of neuronal responses reliably discriminate speech in human auditory cortex. *Neuron* 54 1001–1010 10.1016/j.neuron.2007.06.004 17582338 PMC2703451

[B42] Magri C. Schridde U. Murayama Y. Panzeri S. Logothetis N. K. (2012). The amplitude and timing of the BOLD signal reflects the relationship between local field potential power at different frequencies. *J. Neurosci.* 32 1395–1407 10.1523/JNEUROSCI.3985-11.2012 22279224 PMC6796252

[B43] Marco E. J. Hinkley L. B. N. Hill S. S. Nagarajan S. S. (2011). Sensory processing in autism: a review of neurophysiologic findings. *Pediatr. Res.* 69 48R–54R 10.1203/PDR.0b013e3182130c54 PMC3086654 21289533

[B44] Morillon B. Lehongre K. Frackowiak R. S. J. Ducorps A. Kleinschmidt A. (2010). Neurophysiological origin of human brain asymmetry for speech and language. *Proc. Natl. Acad. Sci. U.S.A*. 107 18688–18693 10.1073/pnas.1007189107 20956297 PMC2972980

[B45] Murdoch J. D. State M. W. (2013). Recent developments in the genetics of autism spectrum disorders. *Curr. Opin. Genet. Dev.* 23 310–315 10.1016/j.gde.2013.02.003 23537858

[B46] Peelle J. E. Gross J. Davis M. H. (2013). Phase-locked responses to speech in human auditory cortex are enhanced during comprehension. *Cereb. Cortex* 23 1378–1387 10.1093/cercor/bhs118 22610394 PMC3643716

[B47] Pelphrey K. Deen B. (2012). Brain scans need a rethink. *Nature* 491 S20 10.1038/491S20a 23136657

[B48] Peñagarikano O. Abrahams B. S. Herman E. I. Winden K. C. Gdalyahu A. Dong H. (2011). Absence of CNTNAP2 leads to epilepsy, neuronal migration abnormalities and core autism-related deficits. *Cell* 147 235–246 10.1016/j.cell.2011.08.040.Absence 21962519 PMC3390029

[B49] Peñagarikano O. Geschwind D. H. (2012). What does CNTNAP2 reveal about autism spectrum disorder? *Trends Mol. Med*. 18 156–163 10.1016/j.molmed.2012.01.003 22365836 PMC3633421

[B50] Pu D. Shen Y. Wu J. (2013). Association between MTHFR gene polymorphisms and the risk of Autism spectrum disorders: a meta-analysis. *Autism Res*. 6 384–392 10.1002/aur.1300 23653228

[B51] Rojas D. C. Singel D. Steinmetz S. Hepburn S. Brown M. S. (2013). Decreased left perisylvian GABA concentration in children with autism and unaffected siblings. *Neuroimage* 86 28–34 10.1016/j.neuroimage.2013.01.045 23370056 PMC3773530

[B52] Rotstein H. G. Pervouchine D. D. Acker C. D. Gillies M. J. White J. A. Buhl E. H. (2005). Slow and fast inhibition and an H-current interact to create a theta rhythm in a model of CA1 interneuron network. *J. Neurophysiol.* 94 1509–1518 10.1152/jn.00957.2004 15857967

[B53] Sauseng P. Klimesch W. Gruber W. R. Birbaumer N. (2008). Cross-frequency phase synchronization: a brain mechanism of memory matching and attention. *Neuroimage* 40 308–317 10.1016/j.neuroimage.2007.11.032 18178105

[B54] Schroeder C. E. Lakatos P. Kajikawa Y. Partan S. Puce A. (2008). Neuronal oscillations and visual amplification of speech. *Trends Cogn. Sci.* 12 106–113 10.1016/j.tics.2008.01.002 18280772 PMC3987824

[B55] Schwarzkopf D. S. Anderson E. J. de Haas B. White S. J. Rees G. (2014). Larger extrastriate population receptive fields in autism spectrum disorders. *J. Neurosci.* 34 2713–2724 10.1523/JNEUROSCI.4416-13.2014 24523560 PMC3921434

[B56] Shamir M. Ghitza O. Epstein S. Kopell N. (2009). Representation of time-varying stimuli by a network exhibiting oscillations on a faster time scale. *PLoS Comput. Biol.* 5:e1000370 10.1371/journal.pcbi.1000370 PMC2671161 19412531

[B57] Snowling M. J. Gallagher A. Frith U. (2003). Family risk of dyslexia is continuous: individual differences in the precursors of reading skill. *Child Dev.* 74 358–373 10.1111/1467-8624.7402003 12705560

[B58] Stevenson R. A. Segers M. Ferber S. Barense M. D. Wallace M. T. (2014a). The impact of multisensory integration deficits on speech perception in children with autism spectrum disorders. *Front. Psychol.* 5:379 10.3389/fpsyg.2014.00379 PMC4033130 24904448

[B59] Stevenson R. A. Siemann J. K. Schneider B. C. Eberly H. E. Woynaroski T. G. Camarata S. M. (2014b). Multisensory temporal integration in autism spectrum disorders. *J. Neurosci.* 34 691–697 10.1523/JNEUROSCI.3615-13.2014 24431427 PMC3891950

[B60] Tyzio R. Nardou R. Ferrari D. C. Tsintsadze T. Shahrokhi A. Eftekhari S. (2014). Oxytocin-mediated GABA inhibition during delivery attenuates autism pathogenesis in rodent offspring. *Science* 343 675–679 10.1126/science.1247190 24503856

[B61] Uhlhaas P. J. Singer W. (2007). What do disturbances in neural synchrony tell us about autism? *Biol. Psychiatry* 62 190–191 10.1016/j.biopsych.2007.05.023 17631116

[B62] Weschler D. (2000). *WAIS-III: Echelle de L’intelligence de Wechsler Pour Adultes*, ed. Troisième. (Paris: Centre de Psychologie Appliquée).

[B63] Whittington M. A. Cunningham M. O. LeBeau F. E. N. Racca C. Traub R. D. (2011). Multiple origins of the cortical γ rhythm. *Dev. Neurobiol.* 71 92–106 10.1002/dneu.20814 21154913

[B64] Williams D. L. Cherkassky V. L. Mason R. A. Keller T. A. Minshew N. J. Just M. A. (2013). Brain function differences in language processing in children and adults with autism. *Autism Res.* 6 288–302 10.1002/aur.1291 23495230 PMC4492467

[B65] Zion Golumbic E. M. Ding N. Bickel S. Lakatos P. Schevon C. A. McKhann G. M. (2013). Mechanisms underlying selective neuronal tracking of attended speech at a “cocktail party.” *Neuron* 77 980–991 10.1016/j.neuron.2012.12.037 23473326 PMC3891478

