# Supplementary figures and images for: Atypical coordination of cortical oscillations in response to speech in autism
Source: Front Hum Neurosci. 2015 Mar 27;9:171. doi: 10.3389/fnhum.2015.00171 (PMC4376066; doi:10.3389/fnhum.2015.00171)

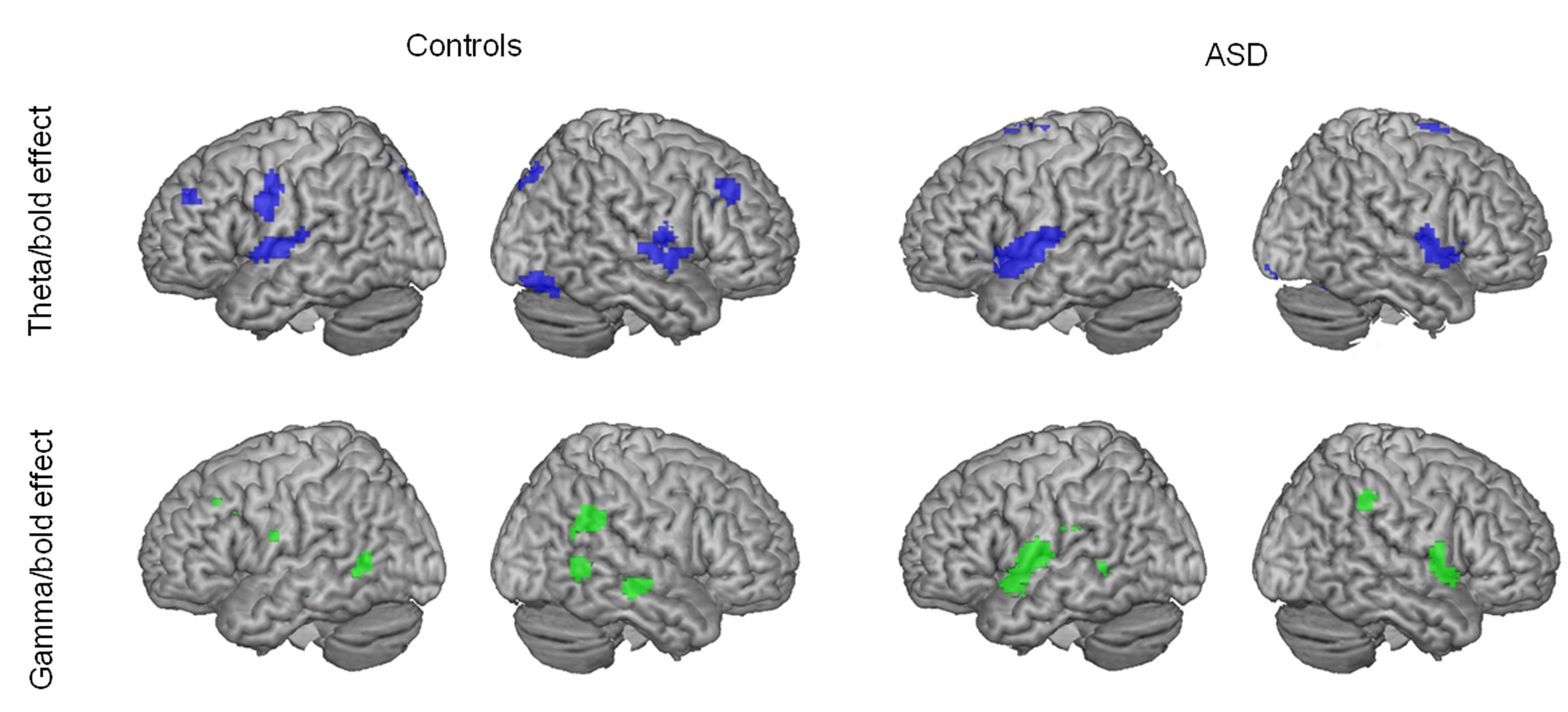

Supplement: Figure S1 — Whole brain topography of theta-BOLD (top) and low-gamma-BOLD (bottom) effects in controls (left) and subjects with autism during the movie (whole brain, p < 0.01 uncorrected; whole brain, p < 0.005 uncorrected; left Heschl’s gyrus p < 0.05 FWE corrected). [file image_1.jpeg]

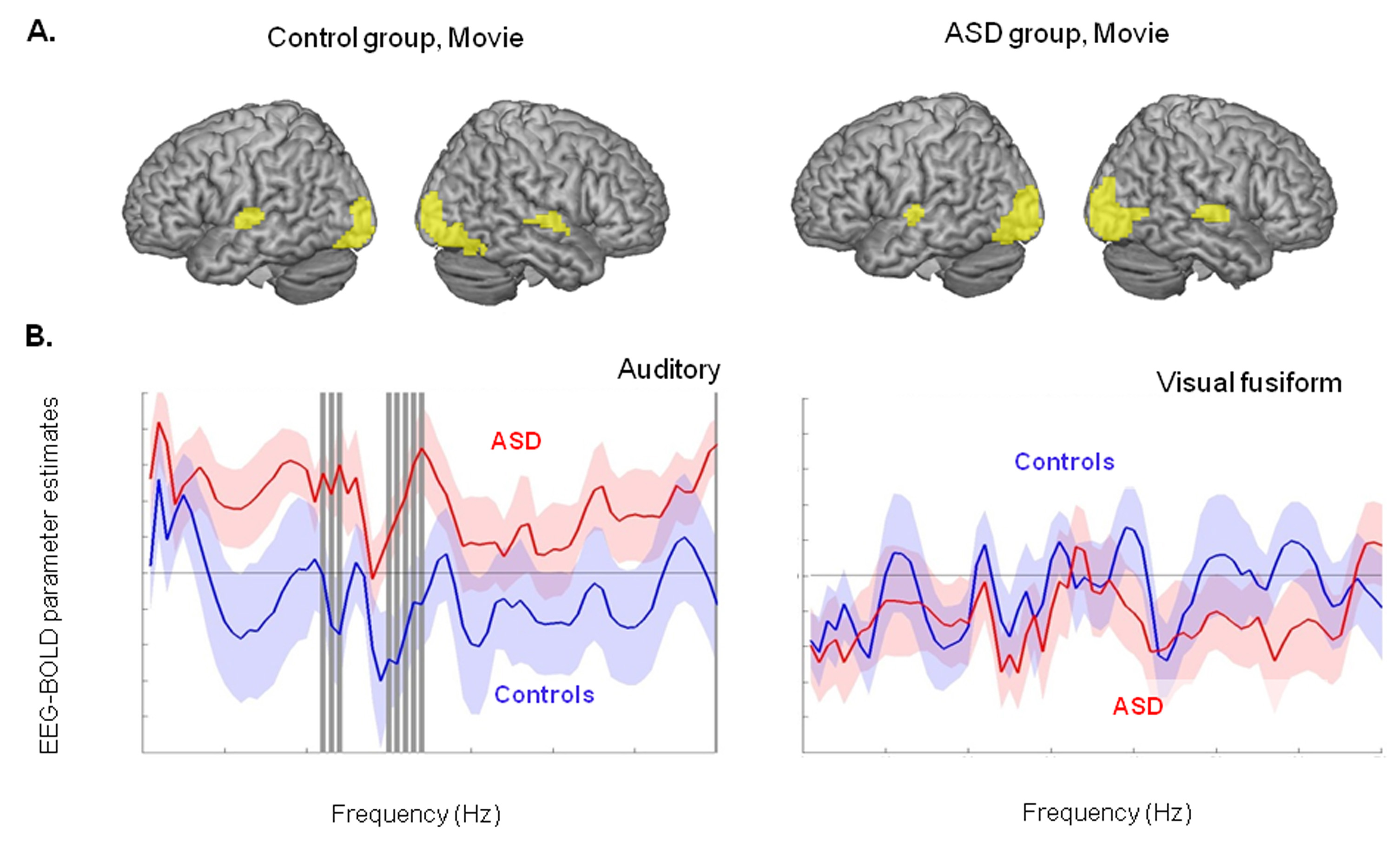

Supplement: Figure S2 — (A) Neural activity (fMRI only, top panels) in subjects with autism (right) and controls (left) during audio-visual presentation of the documentary (p < 0.05, corrected). (B) Partial correlations between the EEG power spectrum (1–70 Hz) and fMRI data at the left hemispheric locations where we found reduced (auditory cortex) and enhanced (visual cortex) neural activity. Note that, unlike the effect in left auditory cortex, the effect in the right posterior superior temporal sulcus is not explained by differences in EEG-BOLD correlations. [file image_2.jpeg]
